# Accurate and efficient protein sequence design through learning concise local environment of residues

**DOI:** 10.1093/bioinformatics/btad122

**Published:** 2023-03-14

**Authors:** Bin Huang, Tingwen Fan, Kaiyue Wang, Haicang Zhang, Chungong Yu, Shuyu Nie, Yangshuo Qi, Wei-Mou Zheng, Jian Han, Zheng Fan, Shiwei Sun, Sheng Ye, Huaiyi Yang, Dongbo Bu

**Affiliations:** Key Lab of Intelligent Information Processing, SKLP, Institute of Computing Technology, Chinese Academy of Sciences, Beijing 100190, China; University of Chinese Academy of Sciences, Beijing 100110, China; Key Lab of Microbial Physiological & Metabolic Engineering, State Key Lab of Mycology, Institute of Microbiology, Chinese Academy of Sciences, Beijing 100101, China; Beijing Advanced Innovation Center for Big Data-based Precision Medicine, School of Engineering Medicine, Beihang University, Beijing 100083, China; Key Laboratory of Big Data-based Precision Medicine (Beihang University), Ministry of Industry and Information Technology of the People’s Republic of China, Beijing 100083, China; Key Lab of Intelligent Information Processing, SKLP, Institute of Computing Technology, Chinese Academy of Sciences, Beijing 100190, China; University of Chinese Academy of Sciences, Beijing 100110, China; Zhongke Big Data Academy, Zhengzhou, Henan 450046, China; Key Lab of Intelligent Information Processing, SKLP, Institute of Computing Technology, Chinese Academy of Sciences, Beijing 100190, China; University of Chinese Academy of Sciences, Beijing 100110, China; Zhongke Big Data Academy, Zhengzhou, Henan 450046, China; Key Lab of Microbial Physiological & Metabolic Engineering, State Key Lab of Mycology, Institute of Microbiology, Chinese Academy of Sciences, Beijing 100101, China; School of Life Sciences, Hebei University, Baoding, Hebei 071002, China; Key Lab of Microbial Physiological & Metabolic Engineering, State Key Lab of Mycology, Institute of Microbiology, Chinese Academy of Sciences, Beijing 100101, China; School of Life Sciences, Hebei University, Baoding, Hebei 071002, China; University of Chinese Academy of Sciences, Beijing 100110, China; Institute of Theoretical Physics, Chinese Academy of Sciences, Beijing 100190, China; Key Lab of Microbial Physiological & Metabolic Engineering, State Key Lab of Mycology, Institute of Microbiology, Chinese Academy of Sciences, Beijing 100101, China; Institutional Center for Shared Technologies and Facilities, Institute of Microbiology, Chinese Academy of Sciences, Beijing 100101, China; Key Lab of Intelligent Information Processing, SKLP, Institute of Computing Technology, Chinese Academy of Sciences, Beijing 100190, China; University of Chinese Academy of Sciences, Beijing 100110, China; Zhongke Big Data Academy, Zhengzhou, Henan 450046, China; Beijing Advanced Innovation Center for Big Data-based Precision Medicine, School of Engineering Medicine, Beihang University, Beijing 100083, China; Key Laboratory of Big Data-based Precision Medicine (Beihang University), Ministry of Industry and Information Technology of the People’s Republic of China, Beijing 100083, China; University of Chinese Academy of Sciences, Beijing 100110, China; Key Lab of Microbial Physiological & Metabolic Engineering, State Key Lab of Mycology, Institute of Microbiology, Chinese Academy of Sciences, Beijing 100101, China; Key Lab of Intelligent Information Processing, SKLP, Institute of Computing Technology, Chinese Academy of Sciences, Beijing 100190, China; University of Chinese Academy of Sciences, Beijing 100110, China; Zhongke Big Data Academy, Zhengzhou, Henan 450046, China

## Abstract

**Motivation:**

Computational protein sequence design has been widely applied in rational protein engineering and increasing the design accuracy and efficiency is highly desired.

**Results:**

Here, we present ProDESIGN-LE, an accurate and efficient approach to protein sequence design. ProDESIGN-LE adopts a concise but informative representation of the residue’s local environment and trains a transformer to learn the correlation between local environment of residues and their amino acid types. For a target backbone structure, ProDESIGN-LE uses the transformer to assign an appropriate residue type for each position based on its local environment within this structure, eventually acquiring a designed sequence with all residues fitting well with their local environments. We applied ProDESIGN-LE to design sequences for 68 naturally occurring and 129 hallucinated proteins within 20 s per protein on average. The designed proteins have their predicted structures perfectly resembling the target structures with a state-of-the-art average TM-score exceeding 0.80. We further experimentally validated ProDESIGN-LE by designing five sequences for an enzyme, chloramphenicol *O*-acetyltransferase type III (CAT III), and recombinantly expressing the proteins in *Escherichia coli*. Of these proteins, three exhibited excellent solubility, and one yielded monomeric species with circular dichroism spectra consistent with the natural CAT III protein.

**Availability and implementation:**

The source code of ProDESIGN-LE is available at https://github.com/bigict/ProDESIGN-LE.

## 1 Introduction

Protein sequence design, the inverse of protein folding, aims to assign an appropriate amino acid type for each residue such that the designed protein can fold into the desired backbone structure (Pabo 1983). Computational approaches to protein sequence design have been widely used in rational protein engineering, including the design of functional enzymes (Siegel et al. 2010; Terán et al. 2020), drugs (Whitehead et al. 2012; Silva et al. 2019), and vaccines (Correia et al. 2010, 2014). Despite the extraordinary advances (Xiong et al. 2020; Anand et al. 2022; Ferruz and Höcker 2022), increasing the efficiency and accuracy of protein sequence design remains a challenge.

Protein sequence design can be accomplished using a concurrent strategy, in which the amino acid types of all protein residues are determined simultaneously. Most approaches of this type seek to exploit the inter-residue distances derived from the target backbone structure. For example, SPROF views the inter-residue distance map as an image with the designed sequence as its caption. In an analogy to the task of image captioning, SPROF applies a convolutional neural network (CNN) to infer protein sequence from inter-residue distance map (Chen et al. 2020). ProteinSolver, another representative approach, uses a deep graph neural network to find a protein sequence that satisfies as many inter-residue constraints derived from the target structure as possible (Strokach et al. 2020). The concurrent strategy, although gains the advantage of high efficiency in inference, might not scale well to the proteins out of the training set.

Protein sequence can also be designed in an iterative fashion—at each iteration step, the residue at a randomly selected position of the target structure is mutated to improve the fitness between the entire protein sequence and the target structure. To measure the fitness, FixBB, the Rosetta sequence design’s fixed backbone protocol (Kuhlman et al. 2003), uses Rosetta energy, which is a human-crafted energy function consisting of dozens of energy terms, including physics-based terms such as van der Waals forces and solvation energy, and knowledge-based terms such as torsion angle preference (Alford et al. 2017). Notably, neural networks have been widely used by this strategy: Anand et al. proposed to learn potential directly from existing protein structures using a 3D-CNN model (Anand et al. 2022); SPIN2 (O’Connell et al. 2018), DenseCPD (Qi and Zhang 2020), and ProDCoNN (Zhang et al. 2020) use deep neural networks to predict the most likely substituting residue type at a position conditioned by its surrounding structural features.

Due to the local nature of the residue–residue interactions within proteins, most of the existing approaches exploit the local environment of a specific residue in a target structure (referred to as *target residue* hereinafter), including geometry features (e.g. the relative position of surrounding residues) and chemical context (e.g. amino acid type of neighboring residues and solvent accessibility) to predict amino acid type for the target residue. For example, DenseCPD and 3D-CNN draw a $20\times 20\times 20{Å}^{3}$ box centered on a target residue as the boundary of its local environment. DenseCPD uses four backbone atoms, i.e. $N,C,C_{\alpha },O$, and $C_{\beta }$ in local environments to predict the most likely amino acid types for target residues, which are then fed into FixBB as additional design constraints, aiming to reduce sequence search space during design. In contrast, 3D-CNN uses all atoms within this box, including both backbone and rotamer atoms. It should be noted that at each iteration step, these approaches rebuild rotamers and evaluate full-atom structures, thus inevitably leading to low design efficiency. While this work was in progress, Liu et al. (2022) reported the ABACUS-R approach, which is similar to our approach, differing in the features used to describe local environment.

**Figure 1:**
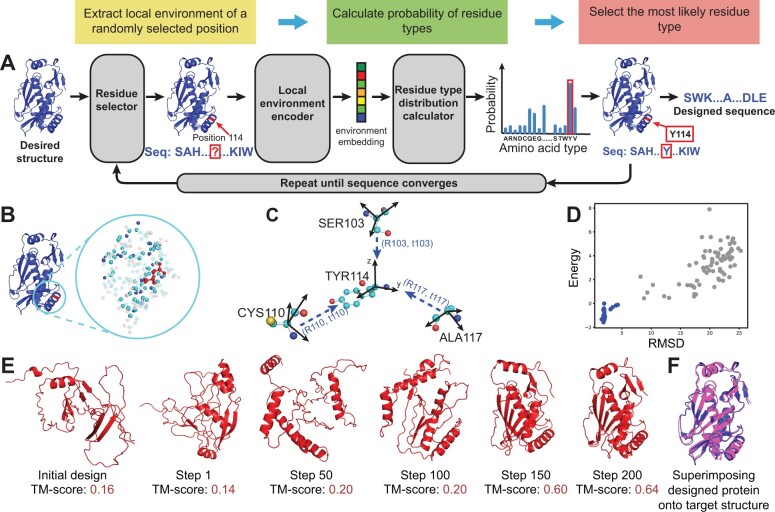
(color online）Overview of protein sequence design process using ProDESIGN-LE. (A) To design a sequence for a desired target backbone structure, ProDESIGN-LE starts from a random sequence and then iteratively assigns an appropriate residue type for a randomly selected position according to its local environment. For example, the local environment of position 114 is fed into the local environment encoder and residue type distribution calculator, yielding a distribution over 20 residue types. ProDEISGN-LE assigns position 114 with the most likely amino acid TYR and thus mutates the design sequence accordingly. ProDESIGN-LE repeats these steps until all residues fit well with their local environments, eventually acquiring a designed sequence. (B) An example of the full-atom local environment around a target residue (in red, or center of the circle), which contains all atoms within a sphere with a predefined radius centered at the residue. (C) The concise but informative representation of the local environment used by ProDESIGN-LE considers the relative positions of neighboring residues. For the residue TYR114, its three neighbors SER103, CYS110, and ALA117 are shown here. For each residue, we construct a local frame with *x* and *y* being the result of applying the Gram–Schmidt process to $\left\{\vec{C_{\alpha }C},\vec{C_{\alpha }N}\right\}$, and *z* being x×y. We then calculate a 3 × 3 transform matrix *R* and a 3D translation vector *t* for each neighbor of TYR114. (D) Energy versus RMSD plot of the predicted structure for the intermediate sequences during redesigning protein CAT III. Here, RMSD measures the proximity of the predicted structure to the target structure. The structures with smaller RMSD usually have lower energy, especially for the native-like proteins with RMSD <5 Å (blue/dark dots). (E) The design process for protein CAT III. The initial random sequence has its predicted structure deviating greatly from the target structure (TM-score: 0.16). After 200 rounds of iteration, ProDESIGN-LE acquires a design with the associated structure perfectly matching the target structure (TM-score: 0.64). (F) The superimposition of the predicted structure for the designed sequence (pink/light) with the target structure (blue/dark)

Here, we present ProDESIGN-LE, an accurate and efficient approach to protein sequence design (Fig. 1A). The rationale underlying our approach is that a designed protein, if every composing residue fits well with its local environment defined by the target structure, is expected to fold into a structure globally resembling the target structure. ProDESIGN-LE uses a concise but informative representation of local environments, which describes the chemical context of a target residue using residue type rather than atoms of neighboring residues. We also use the rotations from a target residue to its neighboring residues as a critical component of a local environment’s representation (Fig. 1C and Supplementary Fig. S1), which have proven to be effective by previous studies on protein structure prediction (Yang et al. 2020; Gong et al. 2021; Jumper et al. 2021).

We further designed a transformer to learn the dependency of a residue on its local environment. ProDESIGN-LE iteratively applies the trained transformer to assign an appropriate residue type for a randomly selected position of the target structure, and updates the local environments of the neighboring residues accordingly, eventually acquiring a designed sequence with all residues fitting well with their local environments. Our approach does not require frequent rebuilding full-atom structures at the intermediate design steps, thus greatly improving design efficiency. In addition, using the residue types to characterize the chemical context, our approach achieves higher accuracy than design approaches considering backbone atoms alone.

We assessed ProDESIGN-LE *in silico* using 68 naturally occurring proteins and 129 hallucinated proteins and compared its performance with FixBB, 3D-CNN, and ProteinSolver. We further experimentally validated ProDESIGN-LE by designing five sequences for an enzyme, chloramphenicol *O*-acetyltransferase type III (CAT III) and recombinantly expressing the designed proteins in *Escherichia coli*. The *in silico* assessing and experimental characterizing results clearly demonstrate the accuracy and efficiency of ProDESIGN-LE in protein sequence design.

## 2 Approach

We aim to design a sequence *S* that can fold into a desired backbone structure *B*, specified using coordinates of each residue’s three backbone atoms, N,Cα, and *C*. Formally, the *i*th residue’s position is represented as $B_{i}=(N_{i},C\alpha_{i},C_{i})$, where $N_{i},C\alpha_{i},C_{i}\in R^{3}$ denote the 3D coordinates of these atoms, respectively. The designed sequence is denoted as $S=s_{1}s_{2}\cdots s_{n}$, where *s_i_* and *n* denote the *i*th residue’s residue type and sequence length, respectively.

We represent the fitness of the designed sequence *S* with the desired backbone structure *B* as a conditional probability P(S|B), which is approximated using the fitness of each residue with its local environment. The local environment of a residue describes its chemical context and geometric features using its neighbors’ residue type and relative positions, respectively. Formally, we define the *i*th residue’s local environment as ${\text{env}}_{i}=\{(s_{j},B_{j}⊖B_{i},j-i)|\|C\alpha_{i}-C\alpha_{j}\|\leq T\}$, where *T* represents a distance cut-off (set as 12 Å in the study), and the operator ⊖ calculates the relative position of two residues, including relative rotation and translation. Using relative position with respect to the target residue $B_{j}⊖B_{i}$ rather than the original coordinate *B_j_*, our approach gains the advantage of rotation and translation invariance. We further represent the fitness of a residue with its surrounding local environment as $P(s_{i}|{\text{env}}_{i})$. Using these notations, we approximate P(S|B) as its pseudo-likelihood:


(1) $$P(S|B)\approx \prod_{i=1}^{n}P(s_{i}|{\text{env}}_{i}).$$

Our design algorithm aims to find a sequence *S* that maximizes the fitness P(S|B). We accomplish this objective by decomposing it into residue-wise subobjectives, i.e. maximizing each residue’s fitness with its surrounding local environment $P(s_{i}|{\text{env}}_{i})$. In particular, our algorithm starts from a random sequence and then iteratively executes the following three steps for improvement: (i) randomly selects a position *i*; (ii) calculates the conditional probability $P(a|{\text{env}}_{i})$ for each possible residue type *a*, and (iii) mutates *s_i_* to be the most likely residue type, i.e. $s_{i}\leftarrow \text{argma}{\text{x}}_{a}P(a|{\text{env}}_{i})$. It should be noted that after mutating *s_i_*, all neighbors of the *i*th residue have their local environments automatically updated. These three steps are iterated until the fitness P(S|B) converges.

In our approach, we learn the conditional distribution $P(s_{i}|{\text{env}}_{i})$ using a transformer as a classifier, which is trained on a subset of the PDB40 dataset (see Section 3.2 for details). The geometrical features in local environment *env_i_*, i.e. the relative position of two residues $B_{j}⊖B_{i}$, is represented as a 3 × 3 rotation matrix together with a 3 × 1 translation vector. We flat the rotation matrix into a 9 × 1 vector and catenate it with the translation vector and other features (e.g. one-hot encoding of residue type and relative sequence position), forming the input feature to the classifier (see Section 3 for details).

Using CAT III protein as an example, we describe the major steps and operations of ProDESIGN-LE below (Fig. 1E). We aim to design a protein with the backbone structure of the CAT III from *E.coli* as the target (PDB entry: 6X7Q, 212 a.a.). Initially, ProDESIGN-LE set the protein sequence randomly as SAHIP…QKIW (the entire sequences of the initial design and intermediate designs are provided in Supplementary Text). As expected, the 3D structure predicted from this random sequence deviates significantly from the target structure (TM-score: 0.16). At the first step, ProDESIGN-LE selected position 114, whose local environment involves the information of its three neighbors, i.e. SER103, CYS110, and ALA117. By feeding this local environment into the geometry transformer and the follow-up fully connected layer, ProDESIGN-LE calculated the probability of all residue types and selected the most likely residue type (TYR: 0.31) to replace the original residue type at this position. After repeating this procedure 200 times, ProDESIGN-LE yielded a designed sequence SWRTVD…SDPE with its predicted structure in perfect agreement with the target structure (TM-score: 0.64). In contrast, the predicted structure of the native sequence achieves a TM-score of 0.66. Figure 1D shows energy of the predicted structures versus their RMSDs with respect to the target structure, which suggests that the structures with smaller RMSD usually have lower energy, especially for the native-like proteins with RMSD <5 Å (blue dots).

## 3 Materials and methods

### 3.1 Network architecture and training procedure

The neural network used by ProDESIGN-LE consists of two major components, including a local environment encoder and a residue type distribution calculator, which are described as follows:

1. *Local environment encoder:* The encoder aims to encode the local environment around a target residue. To accomplish this objective, the encoder uses a transformer with three layers, each layer is composed of a multi-head attention module and a fully connected layer. As performed in Ref. Vaswani *et al.* (2017), we also employ a residual connection around each sub-layer and a follow-up normalization layer (see Supplementary Fig. S1 for details).
2. *Residue type distribution calculator:* The calculator aims to calculate the distribution over all possible 20 amino acid types for a target residue. The calculator uses a fully connected layer followed by a softmax layer to transform the embedding of a local environment into a distribution over residue types.

In the study, the transformer has three layers, where each layer has 16 attention heads, and each head yields an embedding vector of 16 dimensions. We have also tested other settings of these hyperparameters, and we use this setting as ProDESIGN-LE is insensitive to the setting of hyperparameters (see Supplementary Table S1 and Supplementary Text for details). The cross-entropy loss function is used as our model’s optimization objective function. We trained our model with a batch size of 1000 using Adam optimizer ($\beta_{1}=0.9,\beta_{2}=0.999$) with learning rate $1\times 10^{-3}$ (Kingma and Ba 2014). The training process costed about 2 h on a single Nvidia RTX 3090 card.

Here, we use the local structure environment of the target residue at position 114 as an example to illustrate the major steps of the local structure environment encoder and the residue type distribution calculator. As shown in Fig. 1C, the target residue has three residues in its local structure environment, including CYS110, SER103, and ALA117. The encoding process of this local structure environment and the calculation of residue type distribution are as follows:

1. *Feature calculation:* We calculate a 46-dimension feature vector for each of the neighboring residues, which consists of 21 features representing its amino acid type, 12 features representing its position in 3D space relative to the target residue, and 13 features representing its relative position in the sequence.
2. *Feature integration:* We next integrate the features of all three neighboring residues into a 3 × 46 matrix. Then we use a fully connected layer (input dimension: 46; output dimension: 256) to transform each row of the 3 × 46 feature matrix from 46 dimensions to 256 dimensions, thus changing the 3 × 46 feature matrix into a 3 × 256 matrix. In the general case of a target residue with *K* neighbors in its local environment, the feature matrix has a shape of K×46, and the fully connected layer will yield an output matrix with a shape of K×256.
3. *Local structure environment embedding:* We feed the 3 × 256 matrix into a transformer and obtain another 3 × 256 matrix that represents embedding of the three neighboring residues.
4. *Residue type distribution calculation:* We feed the average embedding of the three neighboring residues into a fully connected layer and a softmax layer sequentially, finally acquiring a 20D vector representing the distribution over all possible 20 amino acid types for the target residue. In the present case, TYR has the largest probability of 0.31, and thus we set the target residue as TYR114 and continue the next round of iteration.

### 3.2 Datasets

We trained and tested the transformer using the structures collected in the PDB40 database (Berman et al. 2003). We excluded the structures that contain any DNA/RNA chain or structures that have multiple models. To avoid potential data leaking, we constructed a test set with structures that have no similar structures in the training set; this is achieved via MMseq2 by filtering out the structures in test set with sequences similar to any sequence in the training set (similarity cut-off: e-value $<1\times 10^{-3}$).

Each sample in the training and test set represents a residue of the protein structures thus obtained. In total, we acquired a training set containing 5 867 488 residues extracted from 9995 protein structures and a test set containing 758 160 residues extracted from 401 protein structures. For each residue, we describe its local environment as the following features: (i) the residue type of neighboring residues in one-hot encoding; (2) the relative 3D position of each neighboring residue with respect to the target residue, represented as a rotation matrix and a translation vector, and (3) the distance between a neighbor residue with respect to the target residue in the sequence.

We tested the performance of ProDESING-LE in sequence design using 68 naturally occurring proteins extracted from CASP14 and 129 hallucinated structures (Anishchenko et al. 2021). To avoid potential overlap, we have filtered out the training proteins that are similar to these 68 CASP14 proteins (similarity cut-off: e-value $<1\times 10^{-3}$).

### 3.3 Predicting 3D structure for the designed proteins

We evaluated a designed protein using the proximity between the predicted structure of the designed protein and the target structure. Here, we predict 3D structures for the designed proteins by running AlphaFold2 (Jumper et al. 2021) with its default setting, using templates released before 25 February 2020.

We further applied our in-house software ProFOLD-Single, an improved version of ProFOLD (Ju et al. 2021), to predict structures for the hallucinated proteins. ProFOLD-Single is suitable for assessing designed sequences as it was specially designed for protein structure prediction from a single sequence without requiring any homologous protein.

### 3.4 Protein expression and purification

Coding DNA sequences of designed proteins were constructed into the *BamH I* and *Xho I* sites of pET-28a(+). *Escherichia coli* BL21 (DE3) cells were transformed with the plasmids. Protein expression was induced at an *OD*_600_ between 0.6 and 0.8 with 210 µmol ∕l IPTG for 16 h at 16°C. Cells were harvested and sonicated in a lysis buffer containing 100 mmol/L Tris-HCl and 100 mmol/l NaCl at pH 8.5. The soluble supernatant was purified by Ni-affinity chromatography and collected in pH 8.5 50 mmol/l imidazole buffer. The purified protein was sealed in a dialysis bag at 4°C, replaced with 400 ml 2 mmol/l Hepes buffer for 3 h, and dialyzed three times to avoid Tris and NaCl in the sample.

### 3.5 Analyzing protein thermal stability

The protein thermal stability was analyzed via NanodsF-Prometheus NT.48 device by following the method used in Magnusson et al. (2019). The protein samples were prepared in buffer containing 100 mmol/l NaCl, 10 mmol/l Tris, pH 8.5 with a protein concentration of 0.5 mg/ml. The 10 µl CFEs of samples was loaded in capillaries; the temperature gradient of 1°C/min from 25 to 95° was applied and the intrinsic protein fluorescence at 330 and 350 nm were recorded. The nanoDSC scans were background-corrected and analyzed with Launch NanoAnalyze software.

### 3.6 Circular dichroism spectroscopy

The circular dichroism spectra of the designed CAT proteins and natural CAT protein (PDB entry: 6X7Q) were determined by a Chirascan V100 Circular dichroism spectrometer (Miles et al. 2021). The path length and volume of Quartz cells were 1 mm and 200 µl, respectively. The time-per-point was set as 0.5 s, the scanning step was set as 1 nm, and the scanning ranged from 185 to 280 nm. The spectrum was calibrated with Hepes buffer (2 mmol/l, pH 8.5). The ultraviolet absorbance of CAT protein was measured using a circular dichroism spectrometer, and the absorbance was in the range of 0.6–1.2, indicating that the signal-to-noise ratio was suitable for analysis. The 100 µl samples were loaded at concentrations of 0.15–0.2 mg/ml in pH 8.5 2 mmol/l Hepes buffer into a quartz cell. The results were taken as CD ellipticity in mdeg. The percentages of *α*-helices, *β*-sheets, and random coils of designed CAT proteins and control CAT protein were calculated using the CDNN software with the Net option set as ‘using 23 basespectra’ (advanced CD spectra).

### 3.7 Analytical ultracentrifugation

Sedimentation velocity experiments were performed in a ProteomeLab XL-I analytical ultracentrifuge (Beckman Coulter, Brea, CA) (Hoffmann et al. 2018), equipped with AN-60Ti rotor (4-holes) and conventional double-sector aluminum centerpieces of 12 mm optical path length, loaded with 380 µl sample and 400 µl buffer (10 mmol/L Tris-HCl, pH 7.0, 100 mmol/l NaCl). Before the run, the rotor was equilibrated for approximately 1 h at 20°C in the centrifuge. Then experiments were carried out at 20°C and 41 000 rpm, using continuous scan mode and radial spacing of 0.003 cm. Scans were collected in 3 min intervals at 280 nm. The fitting of absorbance versus cell radius data was performed using SEDFIT software (https://sedfitsedphat.nibib.nih.gov/software/default.aspx) and continuous sedimentation coefficient distribution c(s) model, covering a range of 0–20 S. Biophysical parameters of the buffer are: density $\rho =1.006{g/\text{cm}}^{3}$, viscosity η=0.01031P, and the parameters of proteins are: partial specific volume $V-\text{bar}=0.73000{\text{cm}}^{3}/g$.

All experiments were independently carried out at least three times, and the results were expressed as mean ± standard deviation (SD).

## 4 Results and discussion

### 4.1 Redesign naturally occurring proteins using ProDESIGN-LE

Using 68 naturally occurring protein domains extracted from the CASP14 dataset as representatives (see Supplementary Text for a complete list), we evaluated ProDESIGN-LE and compared it with the widely used design approaches, including 3D-CNN, ProteinSolver, and FixBB. The evaluation criteria include:

1. *sequence matching*: the sequence identity between the designed sequence and the native sequence of the target structure.
2. *structure matching*: the structure similarity (measured using TM-score) between the target structure and the predicted structure from the designed sequence.
3. *sequence–structure matching*: we further evaluated the fitness between the designed sequence and the target structure by building and assessing a threading structure. The threading structure was built as performed by template-based modeling (Eswar et al. 2008), i.e. complementing the target backbone structures with the sidechains determined by designed sequences and then fine-tuning the sidechain conformation using Rosetta relax protocol (Conway et al. 2014). The energy of the obtained threading structure is used as a measurement of the fitness between the designed sequence and the target backbone structure, and an ideal designed sequence is expected to have low energy.

We show the design for protein T1093-D3 as a concrete example (Supplementary Fig. S2). Protein T1093-D3 contains 106 residues with 3 *α*-helices and 10 *β*-strands in its native structure. The sequence identity between the design and protein T1093-D3 is 0.28. For 92 out of the 106 residues, the predicted structure is in perfect agreement with the target structure (mean $C_{\alpha }$ RMSD: 0.84 Å). The designs for 20 proteins are shown in Supplementary Fig. S3 as representatives.

**Figure 2:**
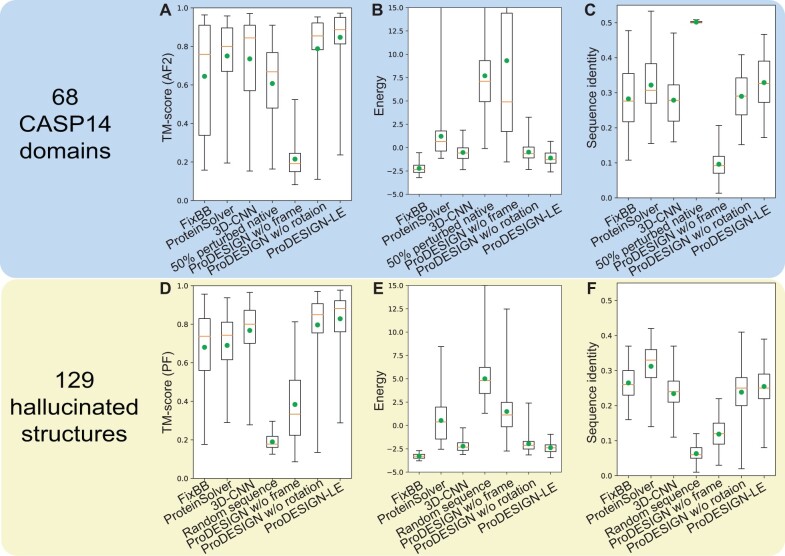
Assessing *in silico* the designed sequences for 68 naturally occurring (A–C) and 129 hallucinated proteins (D–F). We designed sequences for these proteins using FixBB, ProteinSolver, 3D-CNN, and ProDESIGN-LE and assessed the designed sequences using three metrics: (1) the sequence identity between the designed sequence and native sequence of the target structure (C, F); (2) the structure similarity (measured using TM-score) between the target structure and the predicted structure of the designed sequence (A, D); (3) we further built a threading structure through complementing the target backbone structure with the sidechains determined by designed sequences. The energy of the resultant threading structure is used as a measurement of the fitness between the designed sequence and the target backbone structure (B, E). We used AlphaFold2 (AF2) to predict structures for the naturally occurring proteins and ProFOLD-Single (PF) to predict structures for the hallucinated proteins

For the 68 CASP14 proteins, the sequence designs by ProDESIGN-LE achieved a mean sequence identify of 0.33, at the same level as ProteinSolver (0.32), 3D-CNN (0.28), and FixBB (0.28). In contrast, the predicted structures of these designs achieved a mean TM-score of 0.84, which is significantly close to that of the predicted structure using the native sequence (0.88) and higher than all other design approaches, including ProteinSolver (0.75), 3D-CNN (0.74), and FixBB (0.64). In addition, the threading structures generated using the designs by ProDESIGN-LE have lower energy than other design approaches (Fig. 2A–C).

ProDESIGN-LE starts the design process from random sequences. To investigate the effect of starting sequences, we executed ProDESIGN-LE to design sequences for the 68 CASP-14 target proteins starting from the wild-type sequences. The average TM-score of the predicted structures using the acquired design sequences is 0.82, roughly at the same level as those acquired using random sequences as starting sequences (0.84). In addition, when starting from the wild-type sequences, the average loss of the designed proteins is 0.63, also at the same level as that of the random initialization. Taken together, these results indicate the insensitivity of ProDESIGN-LE to the setting of starting sequences.

We also carried out an ablation analysis of ProDESIGN-LE by evaluating the variants with coordinate frames or rotations of neighboring residues removed from local environments. As shown in Fig. 2A and D, the coordinate frames of neighboring residues are critical information of local environments.

It should be noted that these design approaches contain randomization steps, which usually lead to various designed proteins if running multiple times. For the sake of fair comparison, we executed each design algorithm for each target structure only once to obtain a single designed protein for subsequent comparison.

### 4.2 Assessing the generalization of ProDESIGN-LE to hallucinated proteins

We further assessed the generalization of ProDESIGN-LE using 129 hallucinated proteins, which were created through inverting the neural networks trained to predict structures from sequences (Anishchenko et al. 2021). These hallucinated proteins serve as ideal test data for evaluating the generalization of protein design approaches as they are *de novo* and unrelated to the proteins used to train neural networks.

For the 129 hallucinated proteins, all approaches yielded designs with sequence identity at the same level. However, the structures predicted using ProFOLD-Single from the designs by ProDESIGN-LE achieved an average TM-score of 0.83, which is considerably better than the designs by ProteinSolver (0.69), 3D-CNN (0.77), and FixBB (0.68) (Fig. 2D and F). We repeated this experiment using AlphaFold2 as a prediction tool and achieved similar observations (Supplementary Fig. S4).

In addition, when using the designs by ProDESIGN-LE, the average energy of the resultant threading structures is −238.57, which is close to those generated using FixBB (−**328**.72) and lower than those generated using ProteinSolver (53.25), 3D-CNN (−221.50), and random sequences (500.88). Thus, compared with other approaches, the designs by FixBB and ProDESIGN-LE are more compatible with the hallucinated structures (Fig. 2E).

### 4.3 Efficiency of ProDESIGN-LE for protein sequence design

ProDESIGN-LE also showed considerably high time efficiency: ProDESIGN-LE accomplishes sequence design for CAT III with 212 residues within 25 s on an ordinary GPU (Nvidia RTX 3090, memory: 24 GB), which is significantly faster than FixBB (348 s). For the 68 naturally occurring and 129 hallucinated proteins, ProDESIGN-LE accomplished sequence design within 20 s per protein on average. Supplementary Figure S5 suggests that the running time of ProDESIGN-LE increases quadratically with the length of the target structure as expected.

### 4.4 Assessing the effectiveness of local environment on predicting residue type

An ideal definition of the local environment around a residue should effectively describe the preference of residue types in the environment. To assess the effectiveness of the local environment used by ProDESIGN-LE, we calculated the accuracy of the predicted residue types in each local environment. We also investigated the correlation between prediction accuracy and entropy of the predicted distribution of residue types.

**Figure 3:**
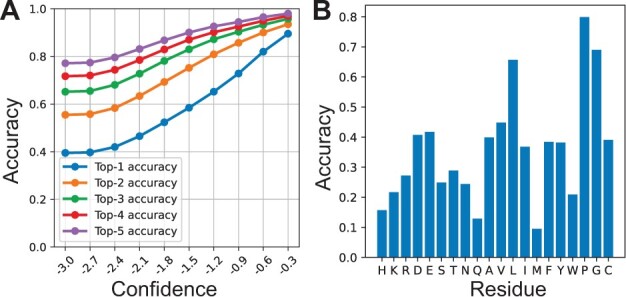
Accuracy of prediction of a residue’s residue type according to its local environment. (A) ProDESIGN-LE predicts a distribution over 20 residue types for a target residue and assigns each residue type with a confidence score. We calculate the top-K (*K* = 1, 2, 3, 4, and 5) accuracy of the predicted residue types exceeding a confidence score cut-off (*x* axis). (B) The relationship between the prediction accuracy and the ground-truth residue type extracted from the native sequence of the protein

We used the negative entropy as the confidence score of the prediction. We sorted all 758 160 local environments extracted from the test set according to their confidence scores and then calculated the accuracy of the predicted residue types that might appear in these local environments. The top 10% of local environments with the highest confidence score achieved a top-1 and top-5 prediction accuracy of 0.902 and 0.982, respectively (Fig. 3A). Even for the top 50% of local environments with high confidence score, the prediction accuracy is still considerably high (top-1 accuracy: 0.567, top-5 accuracy: 0.892).

An in-depth examination suggested that the prediction ability of a local environment is tightly correlated with the amino acid type of the central residue. As shown in Supplementary Fig. S6, the prediction confidence score of local environments exhibits a substantial correlation with the solvent accessibility of target residues (Pearson correlation coefficient: −0.68). Moreover, the prediction accuracy is high in the case that the ground-truth residue is Pro (0.80) or Gly (0.69), implying that local environments around these residues are more constrained (Fig. 3B). This corresponds well with previous studies: proline and glycine confer unique structural constraints on the backbone due to proline’s distinctive cyclic structure and glycine’s lack of side chain (Hayes and Brooks III 2021; Yaron and Naider 1993), thus forming distinctive local environments around these residues. In contrast, the prediction accuracy is relatively low in the case that the ground-truth residue is Met (0.09): methionine is frequently predicted as leucine (Supplementary Fig. S7), which coincides with its chemical property.

It should be also noted that the prediction accuracy increases monotonically with the confidence score (Fig. 3A), thus enabling the use of the confidence score as an effective index of the reliability of prediction.

### 4.5 Experimental characterization of the designed sequences of CAT III

CAT III is an enzyme that confers resistance of antibiotic chloramphenicol to certain bacterium (Murray and Shaw 1997). The functional form of CAT III is a homotrimer, of which the substrate pockets lie between two adjacent protomers. We chose the CAT III from *E.coli* as the design target, which consists of 212 residues, forming five *α*-helices, two $3_{10}$-helices, and two *β*-sheets (Fig. 1F).

We used CAT III as a representative example to evaluate ProDESIGN-LE by experimentally characterizing its designs for this protein. In particular, we executed ProDESIGN-LE using the backbone structure of CAT III as a target structure and acquired five designed sequences as results (denoted as CAT-h1, CAT-h2, CAT-h3, CAT-h4, and CAT-h5, see Supplementary Text for their sequences). To experimentally characterize the designed sequences, we synthesized their coding genes and recombinantly expressed the corresponding proteins in *E.coli*. The recombinant proteins were separated using SDS-PAGE. As shown in Supplementary Fig. S8, all five proteins were successfully expressed, and matched well with the mass of the 6xHis-tagged protein at around 29 kDa. In addition, three out of the five proteins, CAT-h2, CAT-h3, and CAT-h4, were expressed as soluble forms.

**Figure 4:**
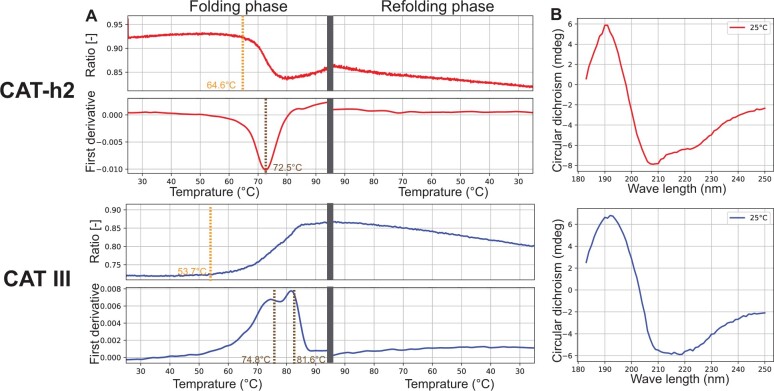
Experimental characterization of the designed protein and natural CAT III protein. (Column A) Thermostable analysis of the two proteins by nanoDSF measurement. The designed protein CAT-h2: the onset denaturation temperature of protein is 64.6°C and the folding Tm value is 72.5°C; The natural protein CAT III: the onset denaturation temperature of protein is 53.7°C and the folding Tm value is 74.8°C. Ratio: 350 nm/330 nm fluorescence intensity. (Column B) Circular dichroism spectra of the proteins from 185 to 260 nm at 25°C. The designed protein CAT-h2 (red) exhibited circular dichroism spectra consistent with the natural protein CAT III (blue)

We further purified the soluble designs by Ni-affinity chromatography for in-depth analysis. Sedimentation velocity experiment of the 6xHis-tagged CAT-h2 yielded a peak at 28.9 kDa, indicating that CAT-h2 exists in a monomeric form with a mass of approximately 25 kDa (Supplementary Fig. S9). The designed CAT-h2 was highly thermostable with a melting temperature of 72.5°C, comparable with CAT III (74.8°C, Fig. 4A and Supplementary Table S2). In addition, structural superimposition of the predicted structure of CAT-h2 and the target structure revealed a 4.02 Å $C_{\alpha }$ RMSD over 212 residues. Here, we examined the design CAT-h2 by circular dichroism (CD) spectroscopy. The CD spectra of CAT-h2 were in good agreement with those acquired from CAT III (Fig. 4B), showing characteristic profiles of α/β proteins (Supplementary Tables S3 and S4).

Together, these results demonstrate that the designed proteins by ProDESIGN-LE can be successfully expressed and fold into stable structures with the desired secondary structures.

## 5 Conclusion

The *in silico* assessments and experimental characterization results presented here for protein sequence design by ProDESIGN-LE have highlighted the special features of learning the concise but effective representation of local environments around residues. The results have also highlighted the superiority of the design paradigm used by ProDESIGN-LE, in which a protein with an expected global structure is designed by iteratively selecting an appropriate residue at a random position to fit well with its local environment. The accuracy and efficiency of ProDESIGN-LE have been demonstrated using both naturally occurring and hallucinated proteins as representatives, along with the experimental characterization of the designed proteins expressed in *E.coli*.

The present ProDESIGN-LE considers the proximity between the structure of the designs with the target structures only but pays no attention to the biochemical characteristics of the designed proteins. At present, of the five designed proteins for CAT III, three showed excellent solubility, but two were insoluble. One possible reason for the two insoluble proteins might be the overuse of hydrophobic residues on the protein surface (Supplementary Fig. S10). Thus, a possible improvement might be restricting the use of residues in the surface and core. In addition, determining how to design a multimer with significant affinity among subunits is also highly desired. This might be accomplished by adding a neural network module to predict affinity and adding it to the objective function. In future studies, these will be incorporated into ProDESIGN-LE. The algorithm and operations of ProDESIGN-LE can be readily extended to further improve the design success rate without significant modifications of its basic ideas.

ProDESIGN-LE relies heavily on the accuracy of protein structure prediction approaches. For proteins T1033-D1 and T1047s1-D1, even using their native sequences as input, the predicted structures still deviate considerably from the corresponding native structures (TM-score is 0.53 for T1047s1-D1, and 0.42 for T1033-D1), which implies the failure of the structure prediction approaches in these cases. Thus, ProDESIGN-LE will definitely benefit from the continuous progress on protein structure prediction approaches.

Recently, significant progress has been achieved in exploiting the rules underlying protein sequences using the protein language model (Bepler and Berger 2019; Heinzinger et al. 2019; Rives et al. 2021). The use of protein language models will definitely increase the validity of the design sequences, which is another future study.

We anticipate that our work on protein sequence design by ProDESIGN-LE with improved accuracy and efficiency will facilitate rational protein engineering.

## Supplementary Material

btad122_Supplementary_Data

## Data Availability

The target structure underlying this article are available in Protein Data Bank at https://www.rcsb.org, and can be accessed with 6X7Q. The designed seqeunces underlying this article are available in its online supplementary material. Hallucinated structures are available at https://files.ipd.uw.edu/pub/trRosetta/hallucinations2K.tar.gz. CASP14 domains are available at https://predictioncenter.org/casp14/targetlist.cgi.
