# Supplementary material for: Accurate and efficient protein sequence design through learning concise local environment of residues
Source: Bioinformatics. 2023 Mar 14;39(3):btad122. doi: 10.1093/bioinformatics/btad122 (PMC10027430; doi:10.1093/bioinformatics/btad122)
Supplement: btad122_Supplementary_Data [file btad122_supplementary_data.pdf]

Accurate and efficient protein sequence design through learning concise local environment of residues

Bin Huang[1,9,†], **Tingwen Fan**[2,†], **Kaiyue Wang**[3,4,†], **Haicang Zhang**[1,8,9], **Chungong Yu**[1,8,9], **Shuyu Nie**[2,5], **Yangshuo Qi**[2,5], **Wei-Mou Zheng**[6,9], **Jian Han**[2], **Zheng Fan**[7], **Shiwei Sun**[1,8,9], **Sheng Ye**[3,4,✉], **Huaiyi Yang**[2,9,✉], **and Dongbo Bu**[1,8,9,✉]

[1]Key Lab of Intelligent Information Processing, SKLP, Institute of Computing Technology, Chinese Academy of Sciences, Beijing 100190, China
[2]Key Lab of Microbial Physiological & Metabolic Engineering, State Key Lab of Mycology, Institute of Microbiology, Chinese Academy of Sciences, Beijing 100101, China
[3]Beijing Advanced Innovation Center for Big Data-Based Precision Medicine, School of Engineering Medicine, Beihang University, Beijing 100083, China
[4]Key Laboratory of Big Data-Based Precision Medicine (Beihang University), Ministry of Industry and Information Technology of the People's Republic of China, Beijing 100083, China.
[5]School of Life Sciences, Hebei University, Baoding, Hebei 071002, China
[6]Institute of Theoretical Physics, Beijing 100190, China
[7]Institutional Center for Technologies and Facilities, Institute of Microbiology, Chinese Academy of Sciences, Beijing 100101, China
[8]Zhongke Big Data Academy, Zhengzhou 450046, Henan, China
[9]University of Chinese Academy of Sciences, Beijing 100110, China

Correspondence: ***Correspondence should be addressed to Sheng Ye (yesheng@buaa.edu.cn), Huaiyi Yang (yanghy@im.ac.cn) or Dongbo Bu (dbu@ict.ac.cn).*** † ***These authors contributed equally to the study.***

Contents

Supplementary Text 1. Major steps of the design of CAT III 3

Supplementary Text 2. List of the 68 CASP14 domains used to assess ProDESIGN-LE 3

Supplementary Text 3. Designed sequences of CAT III 4

Supplementary Text 4. Robustness test of ProDESIGN-LE 4

Supplementary Table 1. The accuracy of the transformer used by ProDESIGN-LE under various settings of hyper-parameters on validation set 5

Supplementary Table 2. Unfolding temperature Tm of the natural CAT III and the designed CAT-h3 proteins determined by nanoDSF measurement 5

Supplementary Table 3. Secondary structure of the natural CAT III protein determined using circular dichroism spectrometry 5

Supplementary Table 4. Secondary structure of the designed CAT-h2 protein determined using circular dichroism spectrometry 5

Supplementary Figure 1: Architecture of the neural networks used by ProDESIGN-LE 6

Supplementary Figure 2. The predicted structure of the designed sequence by ProDESIGN-LE for protein T1093-D3 7

Supplementary Figure 3. Superimposition of the predicted structures of designed sequences onto the corresponding target structures (20 CASP14 proteins and 20 hallucinated proteins) 8

Supplementary Figure 4. *In silico* assessing the designed sequences for 129 hallucinated proteins 9

Supplementary Figure 5. Running time of ProDESIGN-LE for protein sequence design 10

Supplementary Figure 6. The relationship between the accuracy of the prediction of amino acid type and solvent accessibility of the target residue 11

Supplementary Figure 7. Confusion matrix of the transformer 12

Supplementary Figure 8. SDS-PAGE analysis of the five designed CAT proteins crude extracts 13

Supplementary Figure 9. Sedimentation velocity analysis (SVA) of the designed CAT-h2 protein and the control CAT III protein 14

Supplementary Figure 10. Ratios of hydrophobic, hydrophilic and neutral residues in the core/on the surface of the designed proteins for CAT III 15

Supplementary Text 1. Major steps of the design of CAT III

Initial design (random sequence, 212a.a.):
SAHIPATFHQCASIIPNTWGHSMTDQVKGGKWIKRLSYSFSMCDRFMNDLWDEKPPQWGFTADWNRGSFAKCSHNSMIGDQILFTK
AEKSHYWLILPPICVSSMWPLSVCVQDKKMAAFTLIDYWYSNTNAVEECVCYMTNFSHVQADSGFIDKKIMCRADKKLIELTARWN
MSMESYWVDCHAEFGYICPIRKAYGQCYISYARLWSQKIW
Step 1:
SAHIPATFHQCASIIPNTWGHSMTDQVKGGKWIKRLSYSFSMCDRFMNDLWDEKPPQWGFTADWNRGSFAKCSHNSMIGDQILFTK
AEKSHYWLILPPICVSSMWPLSVCVQDYKMAAFTLIDYWYSNTNAVEECVCYMTNFSHVQADSGFIDKKIMCRADKKLIELTARWN
MSMESYWVDCHAEFGYICPIRKAYGQCYISYARLWSQKIW
Step 50:
SFRTPATFHQCASIIPNTWGHSMTDQVAGGKWLNRLDYSFSLCDRFMNDLWDEKWPQTGFINDINRGSFAVCNDNSMFGDQILFTK
AIYSHKLDVTPPISVSSSWPLDVCVQDYKMAADTYKDNPYSNPQAVEECVCYMTNFSHVQADSGFIDKKPMLRADKHPIELTARPN
MSMEDYWVDCHAQFNYICPIREDYDQYYISYQKLWSQKPW
Step 100:
SWRTPALFLQCDRIIPNTWGHSMPGQVAGTRWLNRLDYYFSLCRRDMNDLFDEVWPQTGFINDNDNFSFAVRNDQLMFFDQILPTV
TVYNPKLDVTPPISWKSSWDLDVCVQDYKMAADIYKDNPYKNPQGVPECVCYMTNFRHDEADSGFIDKKPMLRAQKHPIELTARPN
MSMEDLWVDCHIQFNYECPTQEDVDQWWINYQKLWSSKPE
Step 150:
SWRTVALFLSPDRERYNTWRNSMPGQAAGTRWLNRLDLYFSLSRRDMNLLFYEVYRQTGFINDNDKFSLTVRNDQLMFWDQVLPMV
TIYNPKLDVTPPIQWKSSWDIDEFVRDYKMAADIYKDNPLKVPQGVPEPVCYMTNFVHDRAYSGFEDKKPMLENQKHPIITTARPN
MRGEDLLLDCHIQFNYEVVTQEDVDTYWINYQKLLSSKPE
Step 200:
SWRTVDLFLSPERERYYYYRNIMPGQAAETRWLNRTDLYFSLSRSDMNLLDYEVWRQTLVINDNDKFSLRVRDDQLMSWDRVLPMV
TIRIPKTNTTPPLQWKFSWDINEFVRDYEMALKIYKDNPLKVPQGEPSEVRVMTNFVPDRYYSGFEDKKPNLENQKHPIITYARPN
RVGEDLLLPVSIQFNYAVVTKEDVDTLWINYQKLLLSDPE

Supplementary Text 2. List of the 68 CASP14 domains used to assess ProDESIGN-LE

We assessed ProDESIGN-LE using 68 naturally occurring proteins extracted from the CASP14 dataset shown below.

T1024-D1, T1025-D1, T1026-D1, T1028-D1, T1029-D1,
T1030-D1, T1030-D2, T1031-D1, T1032-D1, T1033-D1,
T1034-D1, T1035-D1, T1037-D1, T1038-D1, T1038-D2,
T1039-D1, T1040-D1, T1043-D1, T1045s1-D1, T1045s2-D1,
T1046s1-D1, T1046s2-D1, T1047s1-D1, T1049-D1, T1050-D1,
T1050-D2, T1050-D3, T1052-D1, T1052-D3, T1053-D2,
T1054-D1, T1055-D1, T1056-D1, T1060s2-D1, T1061-D0,
T1061-D2, T1061-D3, T1065s1-D1, T1065s2-D1, T1067-D1,
T1070-D1, T1070-D2, T1070-D3, T1070-D4, T1073-D1,
T1074-D1, T1078-D1, T1079-D1, T1080-D1, T1082-D1,
T1083-D1, T1084-D1, T1087-D1, T1089-D1, T1091-D1,
T1091-D2, T1091-D3, T1091-D4, T1092-D1, T1092-D2,
T1093-D1, T1093-D2, T1093-D3, T1094-D2, T1095-D1,
T1096-D1, T1096-D2, T1099-D1

Supplementary Text 3. Designed sequences of CAT III

CAT-h1:
EAEEVDVEKDPRREEFEFFRYEHPAGYSITVGLDISYFEEKLKKSDIDKDIFDAHIAALAINKYPNFRLFFKDNKLYRAKTVDPV
IDILNPETNTRIYLSYPFSEDLEEFVKAAEEVLKKYKDDTRLFPEGELPKNYFYISRIPEVVSFSMSLHLADATDDYRPHITFSR
PFRDGDRLILPVSITANLAVLGPSDIAKLVLKIQELAKSDLG

CAT-h2:
EFREVDLENSPRLEEYRYFREEYPSGFTATSFLDITDFLKQLESSDIDKKTALAFVVAKAVNEDPRARLSMKDNKLVVWDKVWPV
VSILDPATNTRSLLWYEFSEDFDEFLKKVEEVLKKYKNDTRLLPQGPLPAAHVYISFTPDAFHTSFTELILDPRDDFSLKIHFGR
PFKEGDRWLLPVSVTANRATQDARTISDLINRIQELLRSDLG

CAT-h3:
EFREVDVENSPRKEELRFFREEYPSGFSATSFLDITDFLKQLESSDIDKKIAEVFVITKAINEEDYFRCAMENDKLVVFDKVNPV
FSIDDPATNTRSFLWFEYSEDFDEFLKNALEVLKKYKNDTRLLPQGPLPENFCFISFTPDVFHTSFTFHILDPRDQYAPVIHFGR
PFKEGDRWLLPVSVTVNRAVADARKISELIRRIQELLRSDLG

CAT-h4:
DWDEADWSLDADAEHMMIFRHVFPHGYTHSYSISSTSRHQMEFDSSTGELNTFFFISIDSLNLDPEMMRWIEENEPIIFTEFWQI
WGFELPEEKVYYYVYFTFSAVGSAFSHNLFKMAQTYADENGQLPQGAYHHYFYLGLGVPRARHTSGAFQSPEAIRLFTQITLTCR
PGEEGVTAFFPWSLEGWAENQDGGHMARIHLTQILRARSELG

CAT-h5:
SFVCVDEKNAWKAQTRRFFRKIAPCGETSSAEIDISMLHMQILKSDYDMSVTARFKRATVINEFPRARKKWYNKRKKVSDKVNLV
DALLLETAEVMGLLWWKHHDDFSSFKDRWDSMVRQYYDDARSYPQGKPPDAFTIHLSIPLIQSTSYAFLIATASDKTYPVLIEWR
NYLKGGSHEKPLSLSVSRWVVTGDSSSVHITDEEQLFNSDLG

Supplementary Text 4. Robustness test of ProDESIGN-LE

To test the robustness of ProDESIGN-LE, we have evaluated ProDESIGN-LE at various settings of its hyperparameters, which are summarized below:

1. Number of transformer layers: when using a transformer consisting of 1, 2, 3, and 4 layers, the accuracy of ProDESIGN-LE (embedding dimension: 16, number of heads: 16) is 37.03%, 40.29%, 41.43%, and 42.25%, respectively. Here, accuracy refers to the transformer's best residue type predicting accuracy on the validation set during training.

2. Embedding dimensions: when setting embedding dimension numbers as 4, 8, 12, 16, and 20, the accuracy of ProDESIGN-LE (number of transformer layers: 3, number of heads: 16) is 41.76%, 41.71%, 41.44%, 41.43%, and 41.39%, respectively.

3. Number of attention heads: when using 4, 8, 12, 16, and 20 attention heads, the accuracy of ProDESIGN-LE (number of transformer layers: 3; embedding dimension: 16) is 41.76%, 41.71%, 41.44%, 41.43%, and 41.39%, respectively.

These results suggest that ProDESIGN-LE is insensitive to the setting of these hyperparameters (Supplementary table 4).

Supplementary Table 1. The accuracy of the transformer used by ProDESIGN-LE under various settings of hyper-parameters on validation set

#layers	#heads	Head dimension	Accuracy
1	16	16	37.03%
2	16	16	40.29%
**3**	**16**	**16**	**41.43%**
4	16	16	42.25%
3	4	16	41.76%
3	8	16	41.71%
3	12	16	41.44%
**3**	**16**	**16**	**41.43%**
3	20	16	41.39%
3	16	4	41.96%
3	16	8	41.42%
3	16	12	41.75%
**3**	**16**	**16**	**41.43%**
3	16	20	41.23%

Supplementary Table 2. Unfolding temperature Tm of the natural CAT III and the designed CAT-h3 proteins determined by nanoDSF measurement

Sample	Onset 1 for Ratio (unfolding)	Inflection Point 1 for Ratio (Unfolding)
CAT III	$53.7 \pm 3.0\,°C$	$74.8 \pm 0.1\,°C$/$81.6 \pm 0.5\,°C$
CAT-h2	$64.6 \pm 0.2\,°C$	$72.5 \pm 0.1\,°C$

Supplementary Table 3. Secondary structure of the natural CAT III protein determined using circular dichroism spectrometry

	180-260 nm	185-260 nm	190-260 nm	195-260 nm	200-260 nm	205-260 nm	210-260nm
α-helix	28.6%	30.6%	29.4%	28.4%	29.0%	28.2%	26.0%
Anti-parallel β-sheet	12.0%	11.0%	12.3%	14.8%	12.6%	13.2%	14.7%
Parallel β-sheet	6.6%	7.2%	7.3%	6.6%	6.2%	5.9%	5.6%
β-Turn	16.2%	15.4%	16.3%	16.6%	16.3%	16.5%	17.2%
Coil	35.8%	37.9%	35.4%	34.1%	34.0%	34.8%	32.9%
Total	99%	102.0%	100.80%	100.5%	98.1%	98.5%	96.5%

Supplementary Table 4. Secondary structure of the designed CAT-h2 protein determined using circular dichroism spectrometry

	180-260 nm	185-260 nm	190-260 nm	195-260 nm	200-260 nm	205-260 nm	210-260 nm
α-helix	19.8%	18.6%	18.7%	18.5%	18.7%	18.9%	19.0%
Anti-parallel β-sheet	21.7%	23.7%	24.1%	24.4%	24.3%	24.3%	24.0%
Parallel β-sheet	5.7%	5.6%	5.5%	5.7%	5.60%	5.6%	5.5%
β-turn	18.3%	18.8%	18.9%	19.0%	19.1%	19.1%	19.0%
Coil	34.9%	34.1%	33.8%	34.3%	34.7%	34.2%	34.3%
Total	100.3%	100.8%	101.0%	101.9%	102.4%	102.1%	101.9%

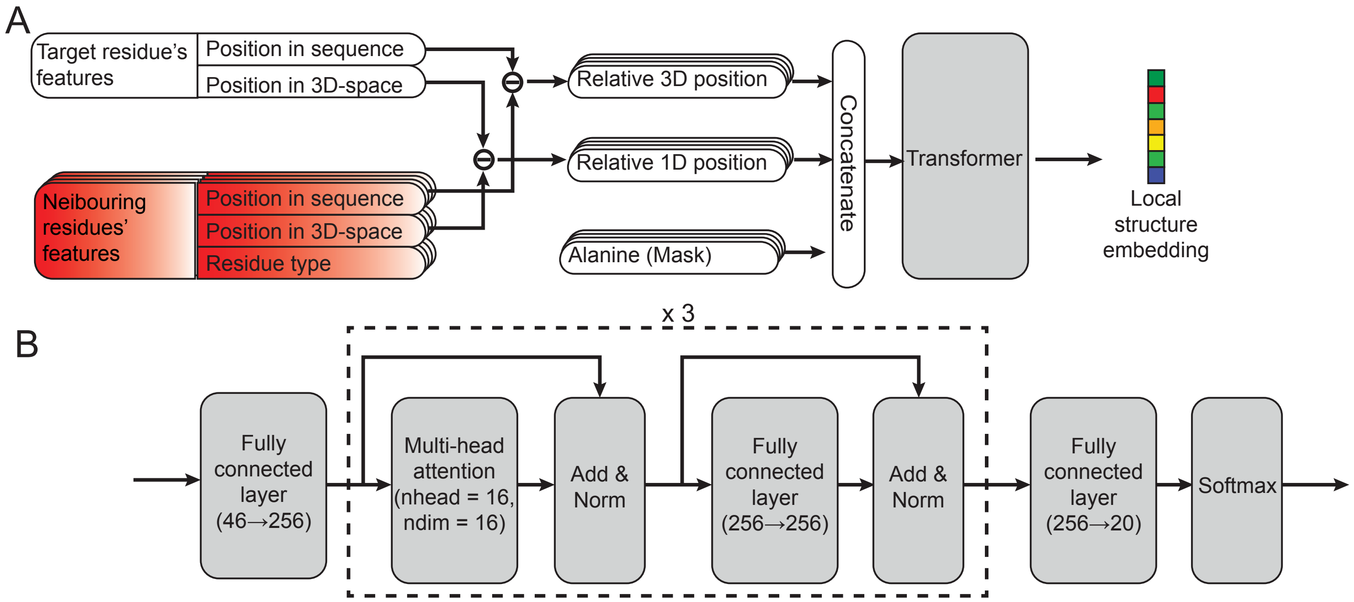

Supplementary Figure 1. Architecture of the neural networks used by ProDESIGN-LE

A, The target residue prediction pipeline of ProDESIGN-LE. **B,** The architecture of the transformer used by ProDESIGN-LE.

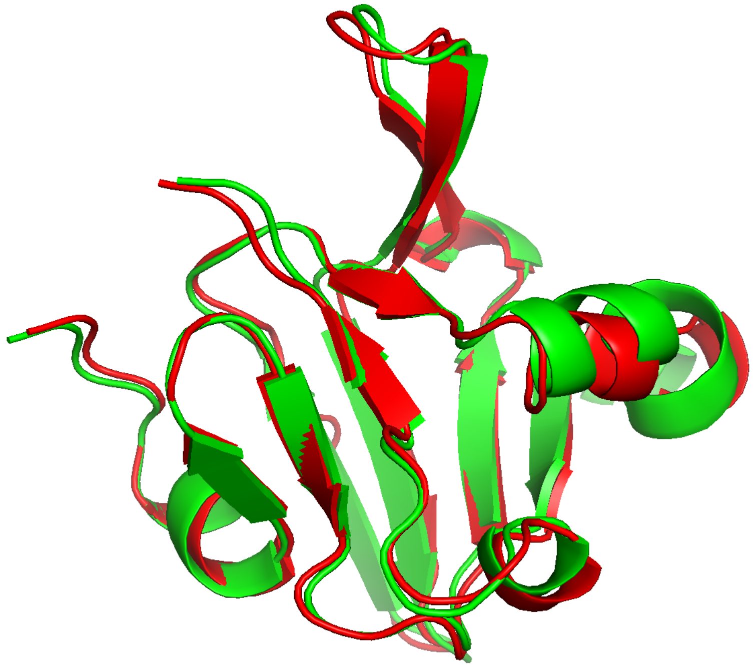

Supplementary Figure 2: The predicted structure of the designed sequence by ProDESIGN-LE for protein T1093-D3

The TM-score between the native structure of protein T1093-D3 (red) and the predicted structure (green) of the designed sequence by ProDESIGN-LE is 0.89.

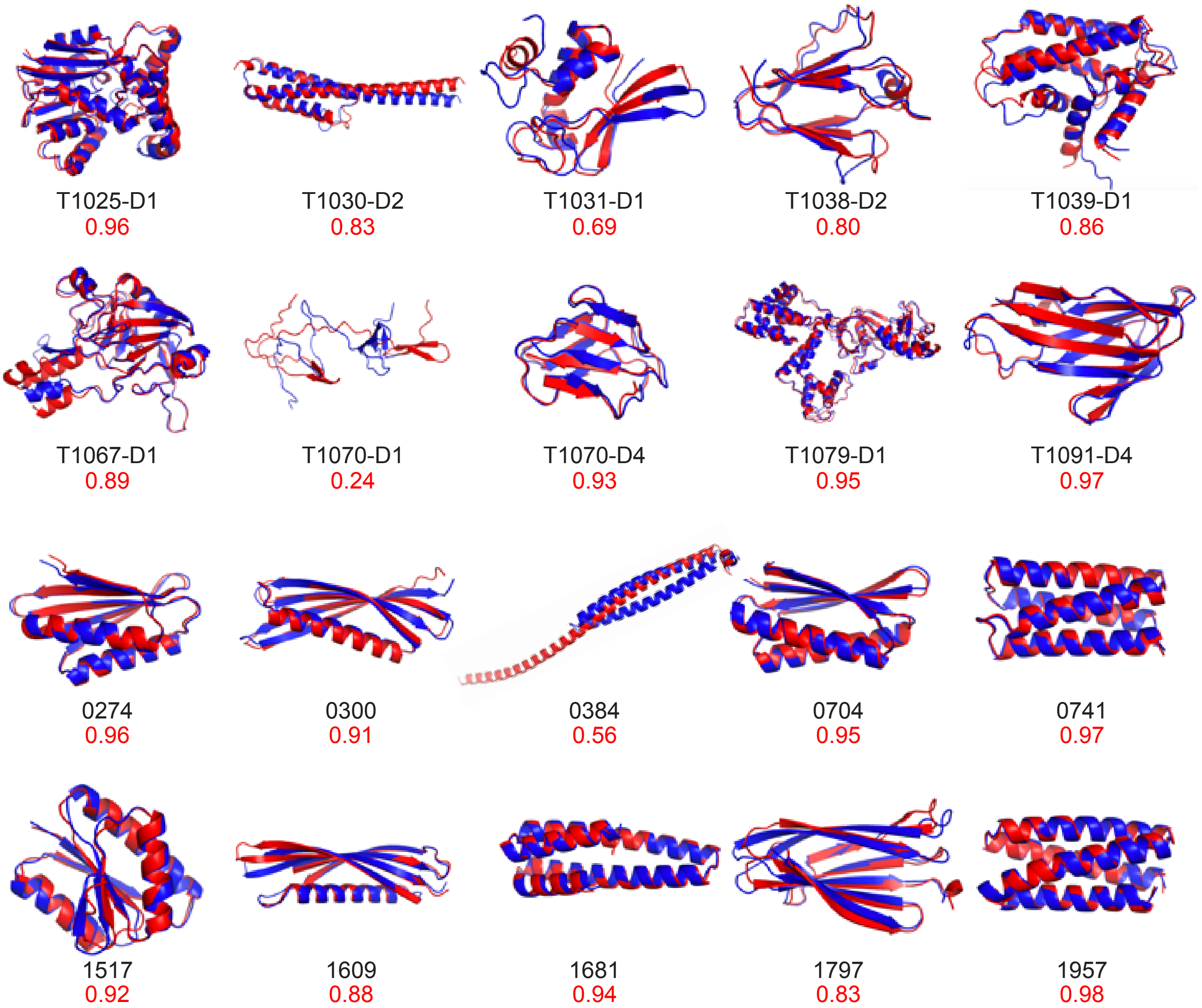

Supplementary Figure 3. Superimposition of the predicted structures of designed sequences onto the corresponding target structures (20 CASP14 proteins and 20 hallucinated proteins)

Ten CASP14 proteins and ten hallucinated proteins are shown here as representatives. The TM-score between the predicted structure (red) and the corresponding target structure (blue) is shown below the superimposition diagram.

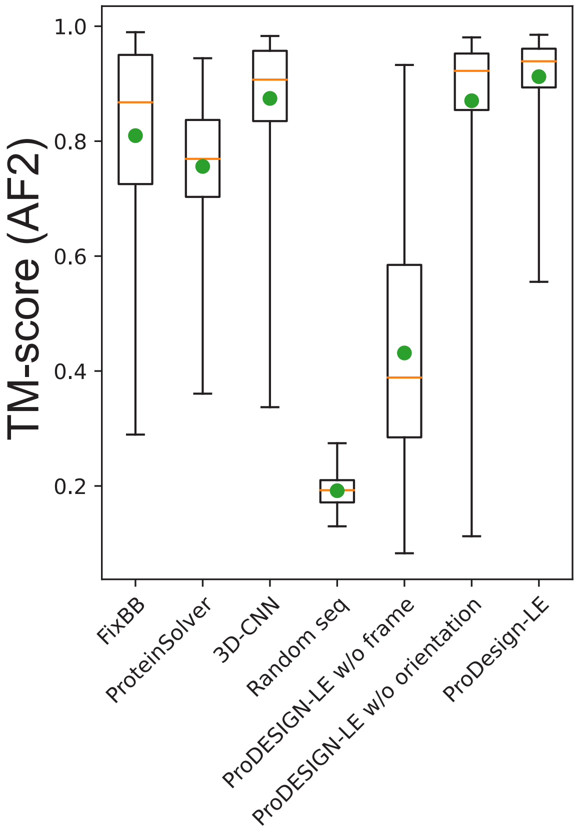

Supplementary Figure 4. ***In silico*** **assessing the designed sequences for 129 hallucinated proteins**

Using AlpahFold2, we predicted structures for the designed sequences of 129 *de novo* hallucinated structures by Rosetta-Fixbb, ProteinSolver, 3D-CNN, and ProDEDIGN-LE. We also predicted the structures using ProFOLD-Single (see Fig. 2D for details).

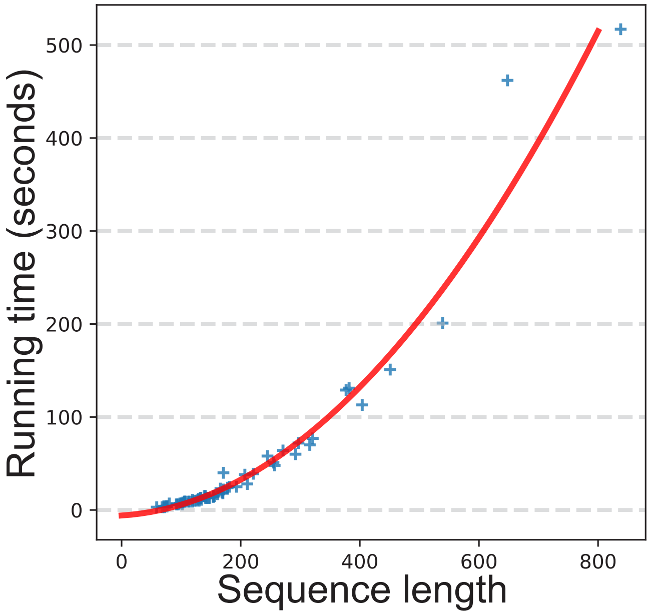

Supplementary Figure 5. Running time of ProDESIGN-LE for protein sequence design

The figure was plotted using the running time of ProDESIGN-LE for design sequences of the 68 CASP14 proteins.

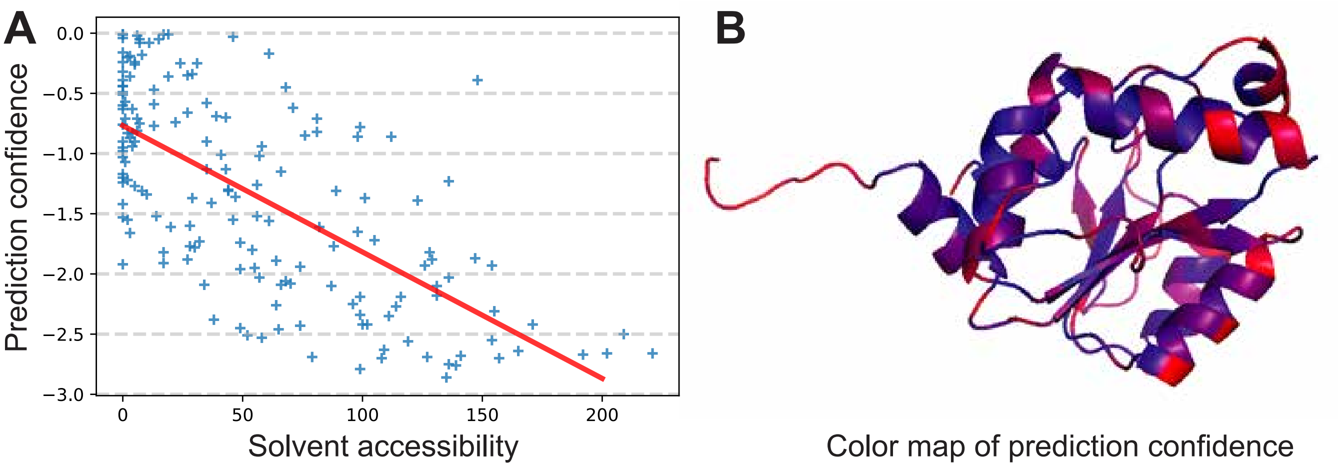

Supplementary Figure 6. The relationship between the accuracy of the prediction of amino acid type and solvent accessibility of the target residue

A, Using CASP14 domain T1045s2-D1 (166 a.a.) as an example, we plot the accuracy of the prediction of amino acid type and solvent accessibility of the target residue (Pearson correlation coefficient: −0.68). **B,** The color map of the prediction confidences on the 166 residues of T1045s2-D1. Red: low confidence, Blue: high confidence

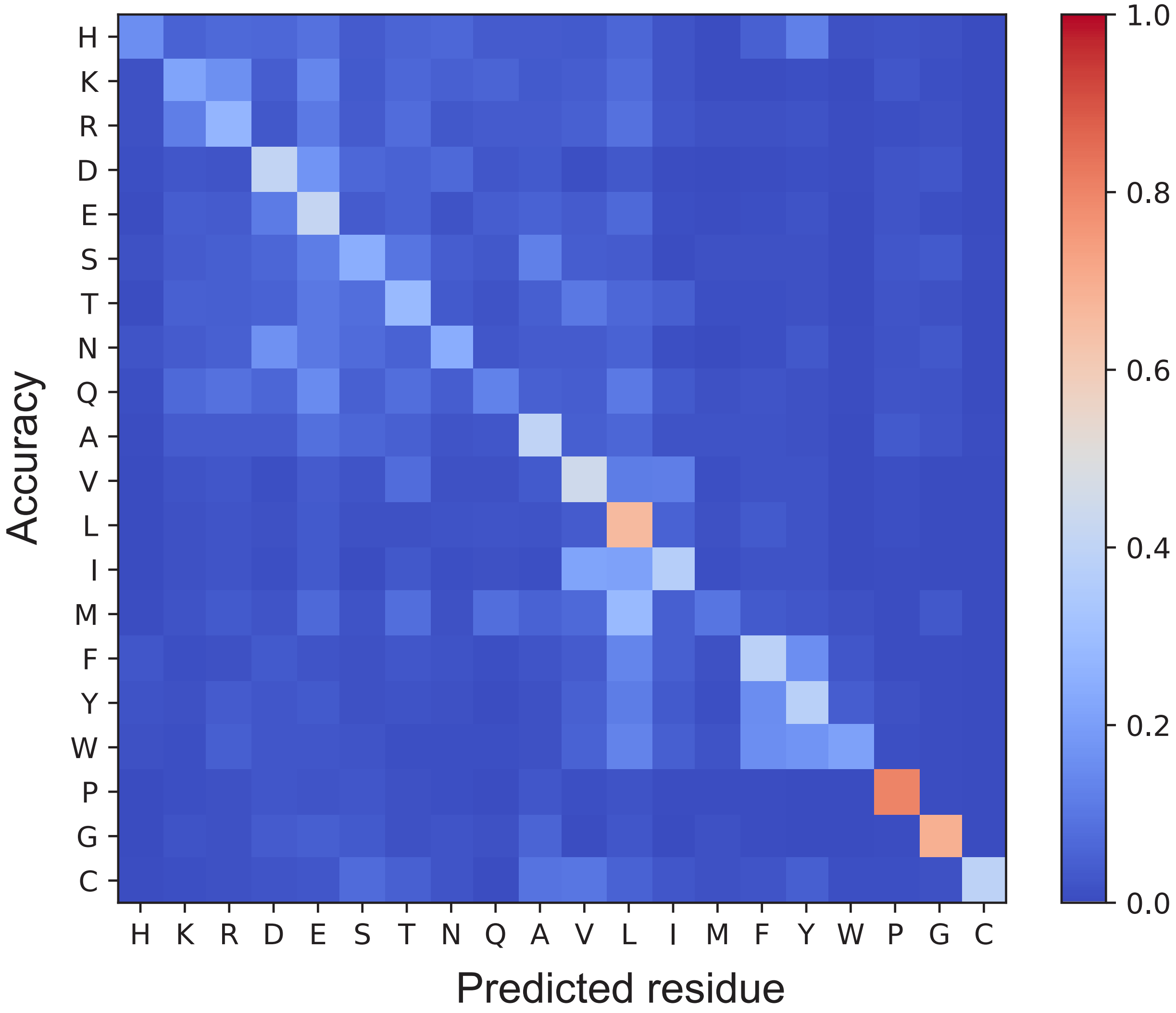

Supplementary Figure 7. Confusion matrix of the transformer

The confusion matrix shows the ground-truth residue type and the predicted distribution over all possible 20 residue.

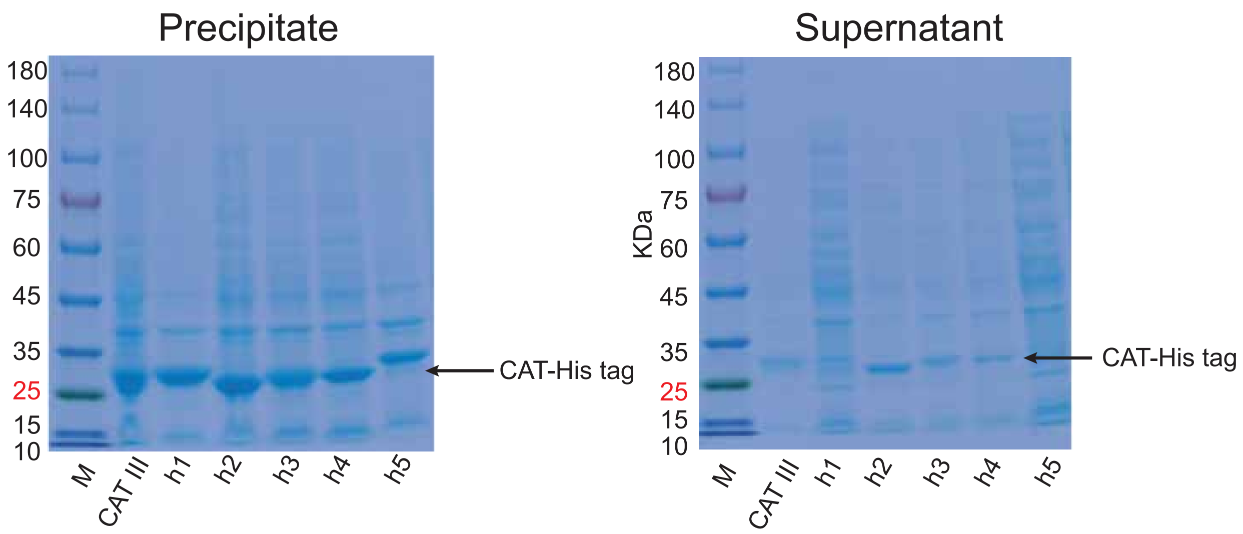

Supplementary Figure 8. SDS-PAGE analysis of the five designed CAT proteins crude extracts

A, SDS–PAGE analysis of the five designed CAT proteins precipitates induced at $16\,^{\circ}\mathrm{C}$.
B, SDS–PAGE analysis of the five designed CAT proteins supernatant induced at $16\,^{\circ}\mathrm{C}$. M: marker; CAT III: The natural protein (PDB: 6x7q). h1-h5: The five designed proteins. Extracted proteins were separated in 4 to $12\,\%$ SDS-PAGE and stained with coomassie blue. Protein samples of $0.1\,\mathrm{OD}$ each were loaded on the gel. The molecular weight of the target protein is $28.3\,\mathrm{kDa}$.

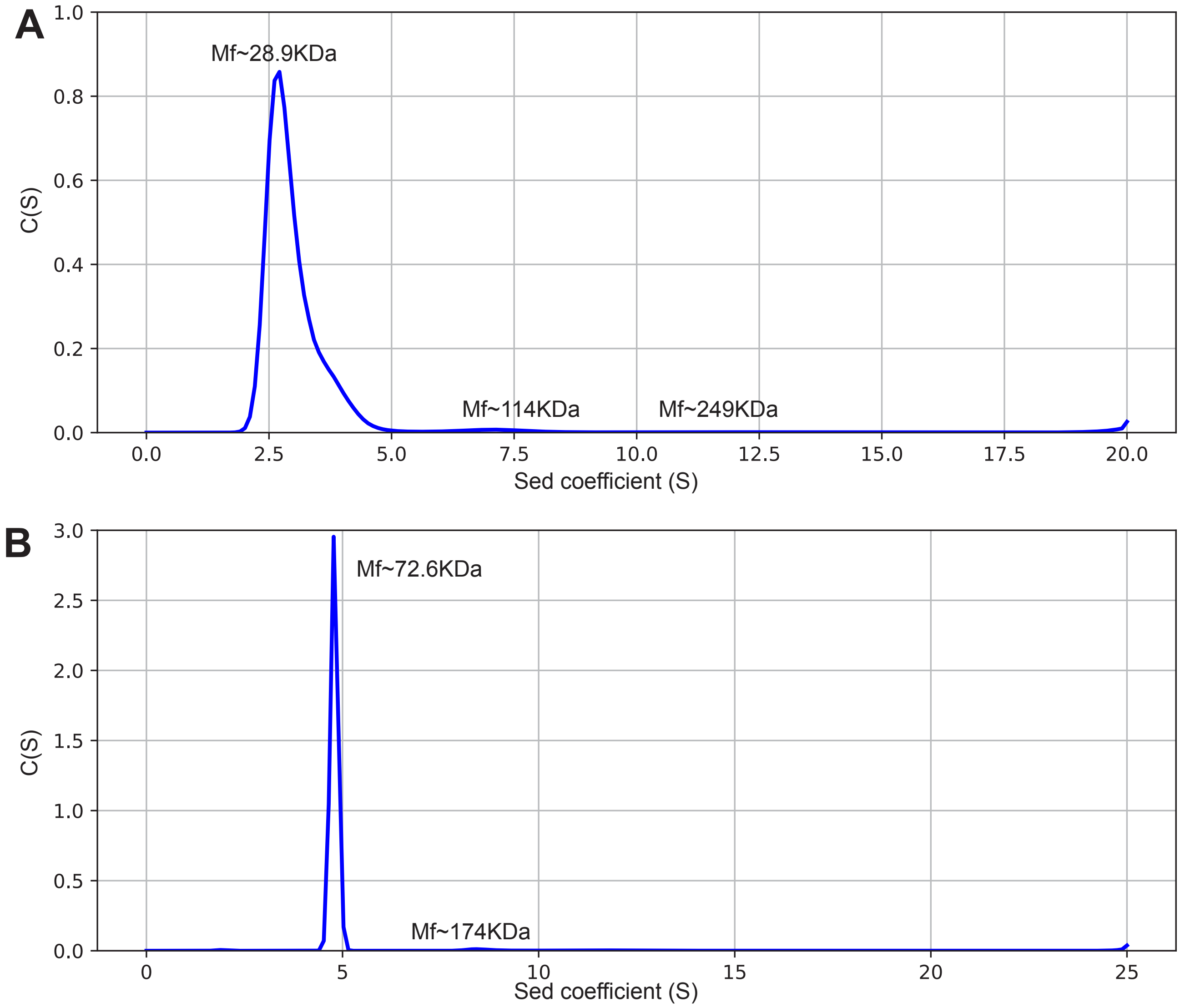

Supplementary Figure 9. Sedimentation velocity analysis (SVA) of the designed CAT-h2 protein and the control CAT III protein

A and B, according to Sedimentation velocity assay methods, obtained the subsidence coefficient (S) of the designed protein of CAT-h2 (A) and the control protein of CAT III (B). **A,** The molecular weight (MW) of CAT-H2 protein ($28.9\,\mathrm{kDa}$) is consistent with the MW of CAT-h2 monomer protein ($28.3\,\mathrm{kDa}$) according to SVA. **B,** MW of control protein of CAT III ($72.6\,\mathrm{kDa}$) is consistent with the MW of control trimer protein ($74.7\,\mathrm{kDa}$) according to SVA.

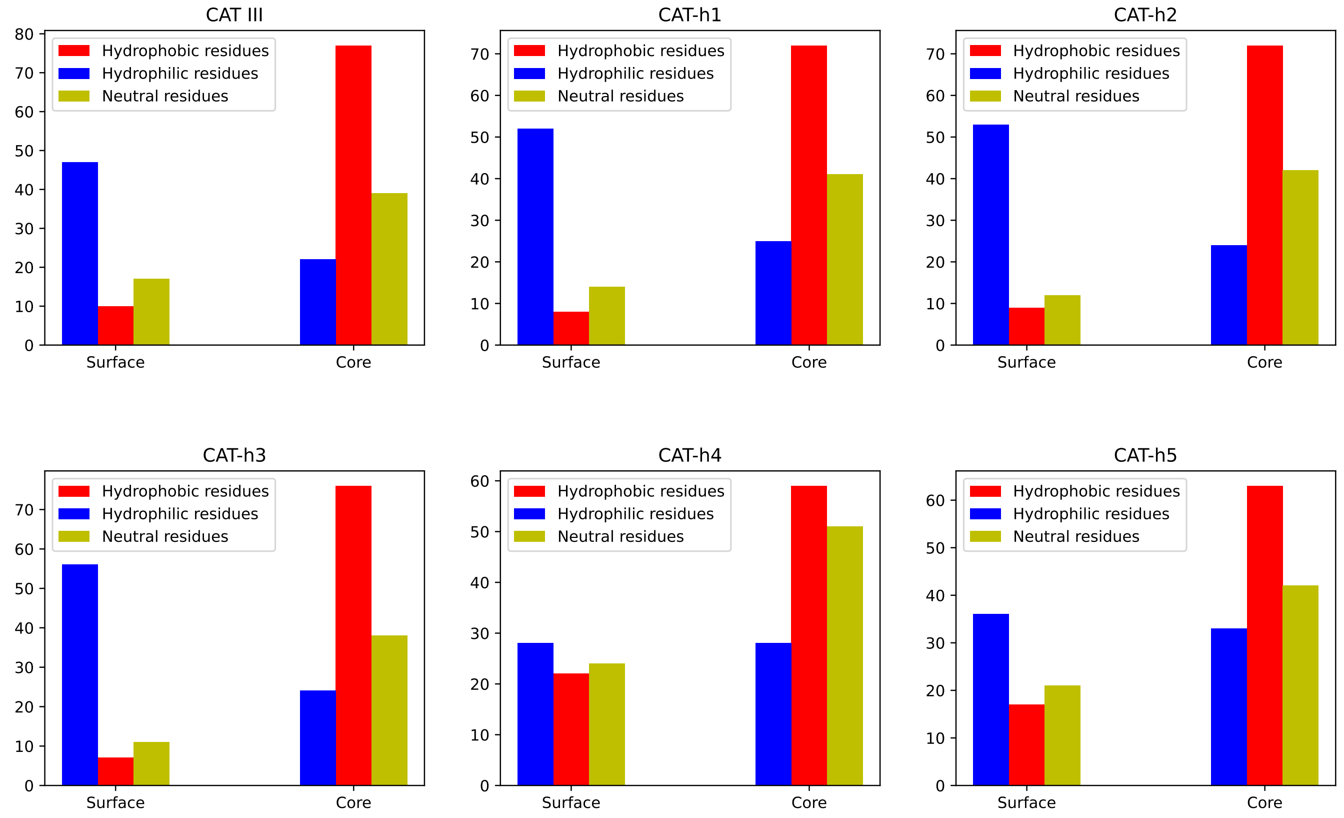

Supplementary Figure 10. Ratios of hydrophobic, hydrophilic and neutral residues in the core/on the surface of the designed proteins for CAT III
